# Sudeck's disease stage 1, or diabetic Charcot's foot stage 0? Case report and assessment of the diagnostic value of MRI

**DOI:** 10.1186/1758-5996-2-60

**Published:** 2010-10-05

**Authors:** Ludger W Poll, Philipp Weber, Hermann-Josef Böhm, Nahid Ghassem-Zadeh, Ernst A Chantelau

**Affiliations:** 1 Department of Radiology, Berufsgenossenschaftliche Unfallklinik Duisburg GmbH, Grossenbaumer Allee 250, D-47249 Duisburg, Germany; 2 Department of Traumatology, Orthopedic and Reconstructive Surgery, Berufsgenossenschaftliche Unfallklinik Duisburg GmbH, Grossenbaumer Allee 250, D-47249 Duisburg, Germany; 3 Diabetes Foot Clinic, Heinrich-Heine-University Düsseldorf, MNR-Klinik, P.O. Box 10 10 07, D-40001 Düsseldorf, Germany; 4 Praxis Dr. Fleischer, Herderstr 71B, D-40237 Düsseldorf, Germany; 5 Holthorster Weg 16, D-28717 Bremen, Germany

## Abstract

**Background:**

The diagnosis of Sudeck's syndrome stage 1 (nowadays termed complex regional pain syndrome I, abbreviated CRPS I) is based on clinical features, namely swelling and pain in a limb. Plain X-ray may be normal. In the absence of pain sensitivity, e.g. in diabetic neuropathy, CRPS I of the foot can be mistaken for Charcot's foot stage 0 (so-called neuro-osteoarthropathy).

**Case presentation:**

The case of a type-1 diabetic woman is reported, in whom CRPS I following a calcaneal fracture was mistaken for Charcot's osteoarthropathy (because of bone marrow edema displayed by conventional MR imaging). In addition, a review is presented on 6 consecutive cases with CRPS I of the foot, and on 20 cases with Charcot's foot stage 0, with particular emphasis on MR imaging findings. The number of bones per foot affected with marrow edema was similar in either condition, with a tendency towards a more patchy, diffuse distribution of bone marrow edema in CRPS I. Bone marrow edema apparently regressed more promptly in response to treatment in Charcot's foot stage 0.

**Conclusion:**

Differentiation of CRPS I from Charcot's foot stage 0 remains a diagnostic dilemma in patients with pain insensitivity. Conventional MRI may be helpful, when repeated for monitoring the treatment response.

## Introduction

The diagnosis of Sudeck's syndrome stage 1 (synonyma: acute Sudeck's disease, warm phase of algodystrophia, complex regional pain syndrome I, CRPS I [1,2]) is based on clinical features, including inflammatory painful swelling and erythema of a limb, preceded by a skeletal trauma in most cases. The case of a woman with type-1 diabetes mellitus is reported, who developed CRPS I following a calcaneal fracture, and in whom this condition was mistaken for diabetic Charcot's foot stage 0 on the basis of MR imaging. This case prompted us to review the effectiveness of conventional MRI in patients with either CRPS I, or Charcot's foot stage 0, respectively.

## Case presentation

**Figure 1:**
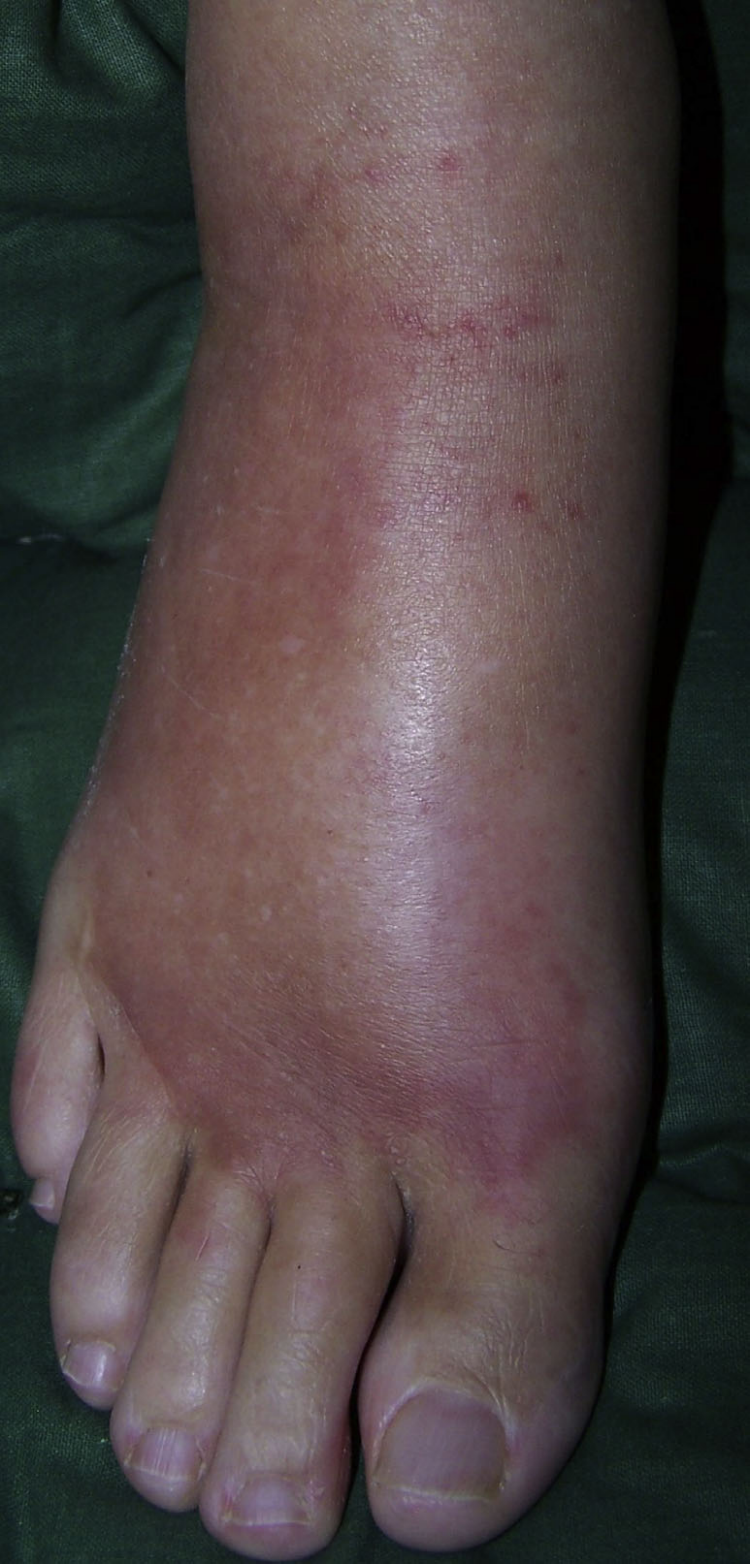
**Appearance of the case patient's right foot: hot, red, swollen, extremely painful without stimulus (CRPS I)**.

**Figure 2:**
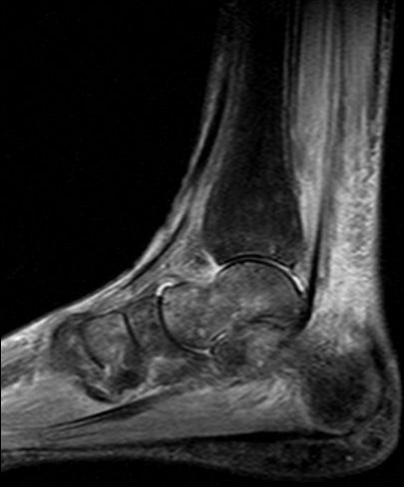
**Same foot as in Figure 1**. MRI sagittal STIR-sequence shows edema of skin and subcutaneous tissue with diffuse and patchy bone marrow edema in the calcaneus, talus, distal tibia and in the anterior tarsus (CRPS I).

**Figure 3:**
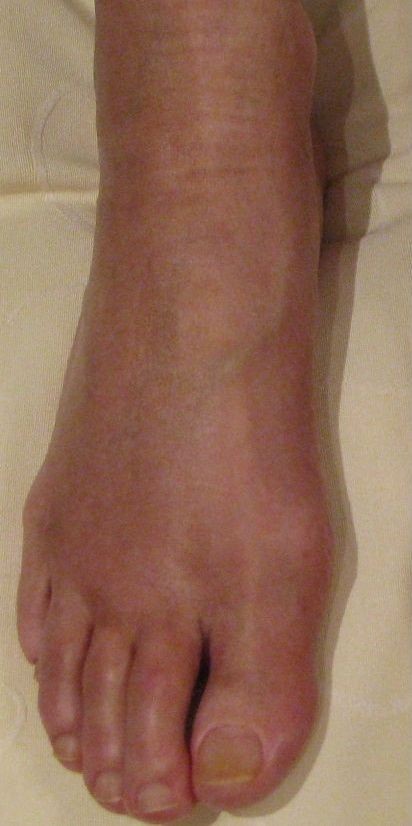
**Same foot as in Figure 1**. 10 months after onset of CRPS I (residual CRPS I).

**Figure 4:**
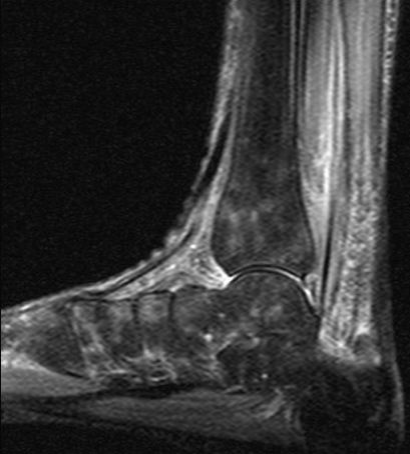
**Same foot as in Figure 3**. Follow-up MRI sagittal STIR-sequence shows patchy regredient bone marrow edema.

A 57-year old female nurse with type-1 diabetes since the age of 12, who was free from any diabetic complications (retinopathy, neuropathy, nephropathy), fell off her bicycle and injured her right foot. She was immediately admitted to hospital where an X-ray was made and the diagnosis of an acute non-displaced calcaneal fracture was established. She was provided with a walking cast and discharged home. Six weeks later, the foot became swollen, hot, and extremely painful. She could no longer walk on it. Repeat X-ray did not show any more calcaneal fracture nor other abnormalities. After 4 more weeks, the patient was referred to the diabetic foot clinic because of presumed Charcot's foot stage 0 [3,4]. On examination, the foot was swollen, warm, erythematous (Figure 1), and extremely painful even to light touch. MR imaging showed soft tissue edema and patchy bone marrow edema in the talus, calcaneus, cuboid and proximal phalanx of the first toe (Figure 2) consistent with neuro-osteoarthropathy (Charcot's foot stage 0, according to the radiologist in charge). The patient was provided with a total contact cast [5], which she could not tolerate; she used a wheelchair instead. Even after 4 weeks of offloading, the foot remained swollen and erythematous- quite unusual for a Charcot's foot. Hence, the diagnosis was revised, and CRPS I was considered instead. The treatment was modified accordingly, and physiotherapy (active and passive foot exercises, warm and cold footbaths) was applied in addition to continuing offloading. Four months later (7 months after onset of CRPS I), swelling and pain had regressed, and gradually increasing load-bearing was advised. Repeat MRI showed fluctuating patchy bone marrow edema, with a tendency to regress. Ten months after onset of symptoms, the patient still had not fully recovered, while the MRI displayed further regression of the bone marrow edema and soft tissue edema (Figures 3 and 4), consistent with improved CRPS I.

## Review of conventional MR imaging

**Figure 5:**
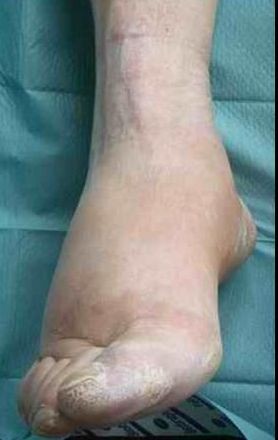
**Example of a Charcot's foot stage 0**. Hot, red, swollen, moderately painful only upon load bearing.

**Figure 6:**
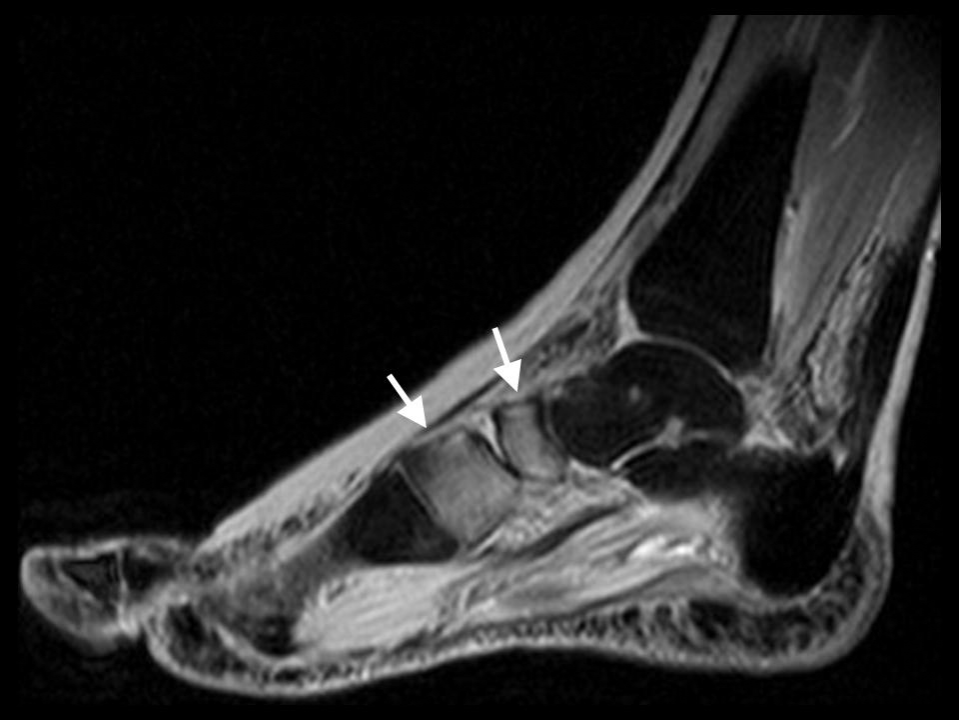
**Same foot as in Figure 5**. MRI sagittal STIR-sequence shows a circumscript edema with skin thickening at the midfoot and the anterior tarsus, with bone marrow edema in the navicular and medial cuneiform bones (white arrows), and edema of adjacent soft tissues (stress injury grade III).

In diabetic patients with insensitive feet due to diabetic neuropathy, CRPS I of the foot [1,2,6-10] may exhibit symptoms similar to acute Charcot's foot stage 0 [3-5] (Figures 1 and 5). Pain as the general symptom is more or less absent, and plain X-ray may be normal in either condition. Other imaging techniques may, thus, be required in those patients. Therefore, we reviewed MRI features in patients with either condition (Figures 2 and 6) in order to assess the diagnostic value of conventional MR imaging.

## Materials and methods

Clinical records of patients with established CRPS I (according to the Budapest criteria [2]) of a foot, under care of the Department of Traumatology, Orthopedic and Reconstructive Surgery at the Berufsgenossenschaftliche Klinik (a tertiary reference centre for vocational injuries in the region of Northrhine-Westphalia) between 2005 and 2010 were reviewed. Likewise, files of patients with established diabetic Charcot's foot stage 0, under care of the diabetes foot clinic at the Heinrich-Heine-University of Düsseldorf/Germany between 1997 and 2007 [11], were reviewed in retrospect. Only cases were included, in whom conventional MRI studies and follow-up data were available.

### Definitions

**Figure 7:**
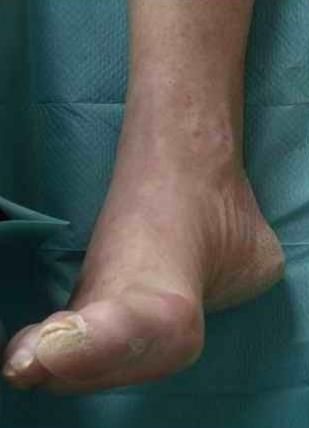
**Same foot as in Figure 5, after 3 months of offloading and immobilisation (healed Charcot foot stage 0, restitution ad integrum)**.

**Figure 8:**
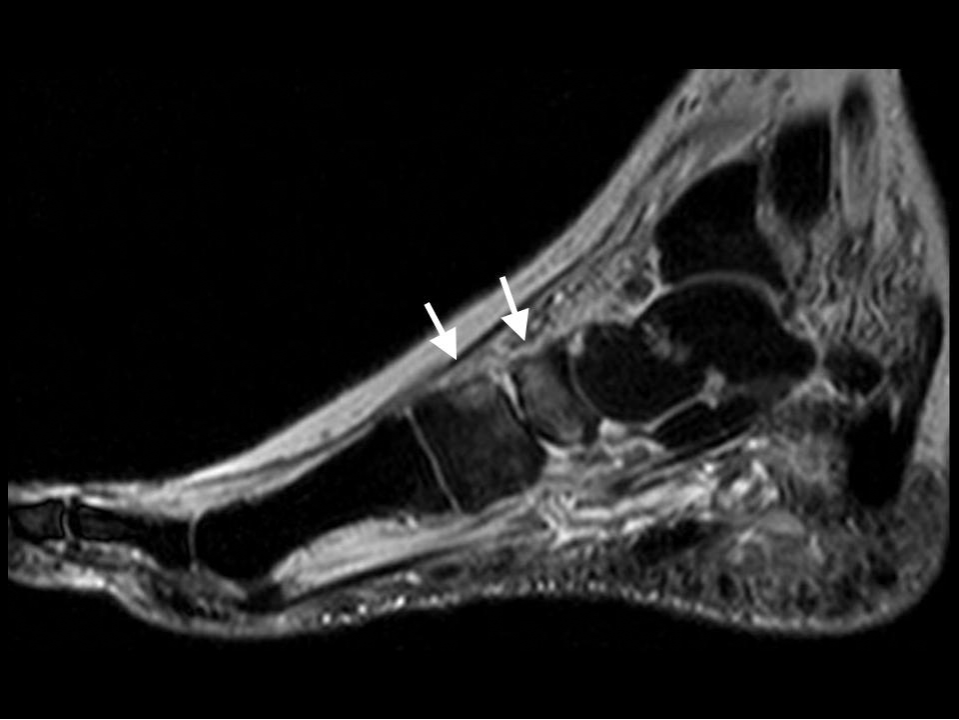
**Same foot as in Figure 7**. MRI sagittal STIR-sequence shows regression of bone marrow edema (white arrows) and soft tissue edema.

CRPS I of the foot was defined by disproportionate painfulness, allodynia, swelling, hyperthermia, erythema and decreased range of motion [1,2]. Diabetic Charcot's foot stage 0 (according to a modified Eichenholtz-scale[4]) was defined by severe sensory neuropathy of the feet (vibration, sensation at the first metatarsal head < 4/8 Rydel-Seiffer tuning fork grade [12,13]), disproportionate painlessness, and swelling, hyperthermia, erythema, decreased range of motion of a foot; plain X-ray had to be normal, and skeletal deformities had to be absent [3-5]. As we have shown previously, this stage of Charcot's foot represents a "silent"-i.e. disproportionally painless- stress injury of the foot skeleton in patients with sensory neuropathy [11-14], see Figures 5,6,7,8.

### MRI studies

Conventional MR examinations (with contrast media, except for 7 cases) were performed on a 1.5 Tesla superconducting magnet. Each foot was scanned in sagittal view using T1-weighted turbo-spin-echo (TSE) sequences (TR: 580, TE: 15) with a slice thickness of 3-4 mm. Paracoronal T1-weighted TSE-sequences were acquired parallel to the midfoot through tibia, calcaneus und talus. Sagittal T2-fat-suppressed-STIR-sequences (TR: 3200, TE: 27, TI 160 msec) were also acquired. Bone marrow edema was defined and charcterized by intermediate to low signal on T1-weighted imaging (T1w).

On fat-suppressed T2-weighted STIR- sequences (short-tau inversion recovery), bone marrow edema appears hyperintense compared to normal low signal intense marrow [15,16].

### Treatment

Patients were treated at the discretion of the physicians in charge. Offloading and immobilisation of the foot by using a total contact cast [5] or wheelchair was applied in cases with Charcot's foot. Various drugs (analgesics, calcitonin, pregabalin, bisphosphonates, vitamin D3, calcium), orthopaedic footwear, crutches and/or physiotherapy were applied in cases with CRPS I.

### Statistics

Data are presented descriptively as means with 95% confidence intervals, unless stated otherwise; Student's t-test was applied. Significance was assumed at a p < 0.05.

## Results

Out of 17 cases of CRPS I of the lower limb referred to the Department of Traumatology, Orthopedic and Reconstructive Surgery, only 6 cases (including the case patient) had CRPS I of a foot. In 5 of these cases, calcaneal fracture was the inciting event, in 1 case it was elective arthroscopic surgery at the talus; first MR imaging was performed 11(95%CI 6-16) weeks thereafter. Out of 40 patients with acute Charcot's foot referred to the diabetic foot clinic [11], 20 cases had Charcot's foot stage 0 and fulfilled the selection criteria. In these patients, first MR imaging was performed 5.5(95%CI 2.7-7.4) weeks after onset of symptoms (p < 0.05 versus patients with CRPS I), showing a stress fracture (grade IV according to Kiuru et al., [14]) of the talus or calcaneus in 3, and of a tarsal or metatarsal bone in 4 patients, respectively, as inciting event. The clinical characteristics of all audited cases are summarised in Table 1. CRPS I patients were younger and free from sensory deficits, whereas all Charcot's patients had polyneuropathy by definition.

**Table 1:** Patient characteristics and MRI features

Patients	CRPS I	Charcot's foot stage 0, no deformity
Total number	6	20
- males/females, n	5/1	10/10
- Age, years	47 (95% CI 40-55)	62 (95%CI 59-65)*
- BMI > 29 kg/m², n	0	4
- Diabetes mellitus, n	1	20**
- Polyneuropathy, n	0	20**
**MRI features**		
Bones with BME per foot, n	3.7(95% CI 1.8-5-5)	4.3(95% CI 3.0-5.6)
Feet with diffuse patchy BME, n	3 (50%)	0 (0%)
Feet with migrating and/or fluctuating BME, n	3 (50%)	0 (0%)
Feet with soft tissue edema, n	5 (83%)	20 (100%)

* p < 0.05; ** by definition. BMI = body mass index; BME = bone marrow edema.

The findings on the conventional MR images are also summarized in Table 1. Charcot's feet responded to treatment by clinical and MRI regression immediately and were healed after 20(95%CI 15-24) weeks. By contrast, CRPS I syndrome fluctuated in symptoms and activity and required significantly longer to heal [43(95% CI 30-76)weeks]; two cases had not healed at the time of writing this report.

## Discussion

The case of our patient demonstrates the diagnostic dilemma that exists in patients with diabetes mellitus and a red, hot, swollen foot with a normal X-ray: clinical symptoms (except for pain quality) as well as MRI findings seem to be suggestive of both, CRPS I, or Charcot's foot stage 0.

CRPS I at the foot is very painful in diabetic and non-diabetic subjects alike: pain is spontaneous, severe, combined with allodynia and hyperalgesia-provided pain sensitivity is normal. By contrast, in diabetic Charcot's foot stage 0 there may be some stress-related pain that subsides upon offloading, but at rest there is little or no spontaneous pain or allodynia- because pain sensitivity is reduced or absent (due to diabetic neuropathy). However, in patients with diabetic neuropathy, CRPS I at the foot may be abnormally painless- and may, therefore, be mixed up with the more common Charcot's foot stage 0.

MRI features of CRPS I (Sudeck's syndrome stage 1) at the foot were demonstrated for the first time by Schimmerl et al. [6]. They showed that in CRPS I bone marrow displays diffuse or spotted hyperintensities ("bone marrow edema") on STIR images, and contrast media enhanced thickening of periarticular and/or subcutaneous and skin tissue. These features, which were confirmed repeatedly [7-10,17] are still not fully understood; increased permeability of small intramedullary and soft tissue vessels due to an unknown stimulus could be causally involved.

MRI features of Charcot's foot stage 0 (in insensitive feet of patients with diabetic neuropathy) were demonstrated previously: subchondral ("traumatic") bone marrow edema, occasionally triggering neuro-osteoarthropathy (Charcot's foot stage I-III) [11-13,18]. In this condition, bone marrow edema is believed to indicate bone or joint contusion, osteitis, or stress-induced trabecular microfracture [14-16,18,19], respectively.

The MRI findings in the present study are not straightforward. In CRPS I, bone marrow edema (and soft tissue edema) seemed to be distributed in a more patchy and diffuse fashion (as compared to the more focal distribution in Charcot's foot stage 0), and to be more fluctuating in response to treatment (in 3 out of our 6 patients, see Figure 2 and 4). It has been reported previously that in CRPS I, bone marrow edema may fluctuate and migrate to neighbouring bones during the natural regression of the acute (warm) phase [7-9]. In Charcot's foot stage 0, however, the bone marrow edema regresses steadily (without fluctuation and migration) in response to strict offloading and immobilisation, like any other traumatic bone marrow edema does [11-14,20], see Figures 6 and 8. Correspondingly, the inflammatory activity-edema, hyperthermia- in a Charcot's foot stage 0 may respond within 3 months of offloading and immobilisation by total contact cast [21], whereas inflammation may fail to respond similarly to the particular therapy in a foot with CRPS I [22].

## Conclusions

In conclusion, although conventional MRI displays distinct skeletal pathology in cases of CRPS I of the foot, and Charcot foot stage 0, respectively, it seems to lack specificity for either condition. Hence, conventional MRI signs have to be interpreted carefully within the clinical context. Further study is warranted to determine the potential of more sophisticated MR imaging techniques like dynamic gadolinium enhanced MRI [23] or diffusion weighted MRI in these conditions.

## List of abbreviations used

BME: bone marrow edema; CRPS: chronic regional pain syndrome; MRI: magnetic resonance imaging

## Consent

Written consent was obtained from the patient P.H. for publication of this case report and any accompanying images. A copy of the written consent is available for review by the Editor-in-Chief.

## Competing interests

The authors declare that they have no competing interests.

## Authors' contributions

LWP conceived the idea for the study and the study design, and drafted the manuscript. PW, HJB, NGZ and EAC provided the patient data. EAC participated in the analysis of the data and the writing of the paper. All authors have read and approved the final version of the article.
